# Alkbh1‐mediated DNA N6‐methyladenine modification regulates bone marrow mesenchymal stem cell fate during skeletal aging

**DOI:** 10.1111/cpr.13178

**Published:** 2022-01-11

**Authors:** Guang‐Ping Cai, Ya‐Lin Liu, Li‐Ping Luo, Ye Xiao, Tie‐Jian Jiang, Jian Yuan, Min Wang

**Affiliations:** ^1^ Department of Endocrinology Endocrinology Research Center Xiangya Hospital of Central South University Changsha China; ^2^ National Clinical Research Center for Geriatric Disorders Xiangya Hospital Changsha Hunan P. R. China; ^3^ Department of Neurosurgery Xiangya Hospital of Central South University Changsha China

**Keywords:** aging, Alkbh1, BMSCs, epigenetic, osteoporosis

## Abstract

**Objectives:**

DNA N6‐methyladenine (N6‐mA) demethylase Alkbh1 participates in regulating osteogenic differentiation of mesenchymal stem cell (MSCs) and vascular calcification. However, the role of Alkbh1 in bone metabolism remains unclear.

**Materials and Methods:**

Bone marrow mesenchymal stem cells (BMSCs)‐specific Alkbh1 knockout mice were used to investigate the role of Alkbh1 in bone metabolism. Western blot, qRT‐PCR, and immunofluorescent staining were used to evaluate the expression of Alkbh1 or optineurin (optn). Micro‐CT, histomorphometric analysis, and calcein double‐labeling assay were used to evaluate bone phenotypes. Cell staining and qRT‐PCR were used to evaluate the osteogenic or adipogenic differentiation of BMSCs. Dot blotting was used to detect the level of N6‐mA in genomic DNA. Chromatin immunoprecipitation (Chip) assays were used to identify critical targets of Alkbh1. Alkbh1 adeno‐associated virus was used to overexpress Alkbh1 in aged mice.

**Results:**

Alkbh1 expression in BMSCs declined during aging. Knockout of Alkbh1 promoted adipogenic differentiation of BMSCs while inhibited osteogenic differentiation. BMSC‐specific Alkbh1 knockout mice exhibited reduced bone mass and increased marrow adiposity. Mechanistically, we identified optn as the downstream target through which Alkbh1‐mediated DNA m6A modification regulated BMSCs fate. Overexpression of Alkbh1 attenuated bone loss and marrow fat accumulation in aged mice.

**Conclusions:**

Our findings demonstrated that Alkbh1 regulated BMSCs fate and bone‐fat balance during skeletal aging and provided a potential target for the treatment of osteoporosis.

## Graphical abstract

Our findings revealed that DNA N6‐methyladenine demethylase Alkbh1 regulates bone marrow mesenchymal stem cell fate during aging. Loss of Alkbh1 inhibited bone formation and promoted marrow fat accumulation. Mechanistically, we identified optn as the downstream target through which Alkbh1‐mediated DNA m6A modification regulated BMSCs fate.

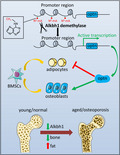

## 1 INTRODUCTION

Age‐related osteoporosis is featured by decreased bone mass and increased bone marrow fat accumulation.^1^, ^2^, ^3^, ^4^, ^5^ BMSCs, which are the common precursors of osteoblasts and bone marrow adipocytes, play a pivot role in keeping bone‐fat balance.^6^, ^7^ Under aging condition, BMSCs tend to differentiate into adipocytes rather than osteoblasts, leading to bone loss and increased marrow fat.^8^, ^9^, ^10^ In the past several decades, Runx2, Osterix, Pparg, and other transcription factors have been identified to regulate BMSCs lineage allocation.^11^, ^12^ Nevertheless, the exact mechanism underlies the switch from osteogenic differentiation to adipogenic differentiation of BMSCs during aging is still unclear. An in‐depth exploration of the molecular mechanisms regulating the balance between adipogenic and osteogenic differentiation of BMSCs will help to develop new strategies to treat osteoporosis.

It is universally accepted that epigenetic control of gene expression, such as DNA methylation, RNA methylation, and histone modifications, is an important pattern of regulating BMSCs fate.^13^, ^14^, ^15^, ^16^, ^17^, ^18^, ^19^ In addition to canonical DNA 5‐methylcytosine (5mC) modification, DNA N6‐methyladenine (N6‐mA) modification has been discovered as a novel type of DNA methylation and acts as a new epigenetic marker in eukaryotic DNA.^20^, ^21^, ^22^ Alkbh1, a 2‐oxoglutarate and Fe^2+^‐dependent hydroxylase, has been identified as a demethylase for DNA N6‐mA.^23^, ^24^ Alkbh1 participates in transcriptional control of trophoblast stem cell marker and plays a vital role in placental trophoblast differentiation.^25^ Alkbh1 is also involved in epigenetic regulation of embryonic stem cell (ESC) gene expression, and depletion of Alkbh1 can lead to an imbalance in ESCs fate decision.^20^ In addition, a study on genomic Alkbh1 knockout mice (Alkbh1^−/−^ mice) showed that most Alkbh1^−/−^ mice died during embryogenesis and deletion of Alkbh1 leaded to reduced ossification and skeletal defects.^26^ Moreover, it has been reported that Alkbh1 can promote osteogenic differentiation of human mesenchymal stem cells (MSCs) and enhance vascular calcification by regulating the level of DNA N6‐mA.^27^, ^28^ Nevertheless, the role of Alkbh1 in bone metabolism is still unclear.

Here, we find that the expression of Alkbh1 decreases in BMSCs from aged subjects. Deletion of Alkbh1 inhibits osteogenic differentiation while enhances adipogenic differentiation of BMSCs.Alkbh1 deficiency in BMSCs results in bone loss and increased marrow fat. Overexpression of Alkhb1 in aged mice promotes bone formation and reduces marrow fat accumulation. Hence, our findings provide a novel insight and potential target for the treatment of osteoporosis.

## 2 MATERIALS AND METHODS

### 2.1 Bioinformatic analysis

Genotype‐Tissue Expression (GTEx) (https://www.gtexportal.org/) is a comprehensive public database for studying human normal tissue‐specific gene expression. We downloaded the expression data of Alkbh1 from the GTEx, analyzed Alkbh1 expression pattern in 31 normal tissues, and generated the expression pattern of Alkbh1.

Gene Expression Omnibus (GEO) database is a public functional genomics data repository. We downloaded microarray data (GSE35955) from the GEO database. The microarray data of GSE35955 included the transcriptome of human BMSCs from 4 elderly people and 5 middle‐aged controls. We used R software (ver. 3.6.3) to analyze the microarray data. The R package was used to normalize the data and analyze the differentially expressed genes (DEGs). The DEGs were screened by limiting log_2_ fold change > 1 and *p*‐value < 0.01. We chose the top 500 DEGs and generate heatmap to reveal these DEGs. Microarray data (GSE30561) were also obtained from the GEO database. The microarray data of GSE30561 included the gene expression profile of Alkbh1 knockout mouse ESCs and wild‐type controls. The heatmap was made to reveal the differences of selected genes associated with BMSCs differentiation.

### 2.2 Mice

To obtain BMSC‐specific Alkbh1 knockout mice, we interbred Prx1‐Cre mice with Alkbh1 flox (Alkbh1^fl/+^) mice to generate Prx1‐Cre; Alkbh1^fl/fl^ mice as conditional Alkbh1 knockout mice. The Alkbh1^fl/fl^ mice were used as controls. We purchased the Prx1‐Cre transgenic mice from Jackson Laboratory and Alkbh1 flox mice from the European Mouse Mutant Archive (EMMA) repository. For BMSC‐specific Alkbh1 knockout experiments, 6–8 male mice per group were used at each observation time point (3 and 15 months). All mice used in this study were maintained in the C57/B6 background.

For Alkbh1 overexpression, adeno‐associated virus expressing the mouse Alkbh1 gene (AAVs‐Alkbh1) was purchased from Hanbio Biotechnology Co. 15‐month‐old male C57/B6 mice received 10 μl AAVs‐ Alkbh1(1 × 10^12^ vg/ml) or control AAVs at the same dosage via intra‐bone marrow injection as described before.^29^ Two months after injection, the mice were sacrificed to analyze the bone phenotypes. Five mice per group were used for each independent experiment.

All mice were housed in the specific pathogen‐free facility of the Department of Laboratory Animals of Central South University.

### 2.3 Cell culture and treatments

Primary BMSCs were isolated from 4‐ to 6‐week‐old mice as described before.^29^, ^30^ The cells were cultured in αMEM containing 100 µg/ml streptomycin, 100 U/ml penicillin, and 15% FBS (Gibco) in a 37°C incubator with 5% CO_2_.

For osteogenic differentiation, BMSCs were cultured in 12‐well plates with osteogenic induction media containing 50 μM ascorbate‐2‐phosphate, 0.1 μM dexamethasone, and 10 mM β‐glycerol phosphate.

For adipogenic differentiation, BMSCs were cultured in 6‐well plates with mouse BMSCs adipogenic induction media (Cyagen Biosciences; MUBMX‐90031).

For plasmid transfection, 1 μg/ml mouse optineurin (optn)‐plasmid (Youbio Biological Technology Co., Ltd) and negative control were transfected into BMSCs with lipofectamine 2000 (Invitrogen) according to the manufacturer's instructions, followed by osteogenic or adipogenic differentiation.

### 2.4 Cell staining

Alkaline phosphatase (ALP) staining was conducted after 7 days of osteogenic induction with a commercial kit (Beyotime Biotechnology; C3206) following the manufacturer's recommendations. Quantitative ALP activity was also detected with an ALP Assay Kit following the manufacturer's protocols (Beyotime Biotechnology; P0321).

Alizarin red staining (ARS) was performed after 3 weeks of osteogenic induction using a commercial ARS solution (Cyagen Biosciences Inc; S0142) following the manufacturer's recommendations. Alizarin red was destained from cell matrix with 10% cetylpyridinium chloride and evaluated by a spectrophotometer at 562 nm.

We performed oil red O staining after 2 weeks of adipogenic induction using a commercial oil red O staining solution (Cyagen Biosciences Inc; S0132) following the manufacturer's instructions. Oil red O was released from lipids into isopropanol and determined by a spectrophotometer at 405 nm.

### 2.5 qRT‐PCR analysis and Western blot

qRT‐PCR analysis and western blot were conducted as previously described.^29^, ^30^, ^31^, ^32^ Table S1 lists the primer sequences for qRT‐PCR in this study. Antibodies for western blot in this study were rabbit anti‐Alkbh1 (Abcam; ab195376); rabbit anti‐αTubulin (Proteintech; 11224‐1‐AP); and mouse anti‐optn (Santa Cruz Biotechnology; sc‐166576).

### 2.6 Dot blotting

Dot blotting was performed as previously reported.^27^ Briefly, the extracted genomic DNA was denatured at 95°C for 10 min, cooled down on ice for 5 min, spotted on the nylon membrane (Millipore), and baked at 80°C for 30 min. Then, we blocked the membrane with 5% milk for 60 min at 25°C. Subsequently, the membrane was incubated with anti‐N6‐mA (Synaptic Systems, 202‐003) at 4°C overnight. The next day, we incubated the membrane with HRP‐linked secondary antibody at 25°C for 60 min, followed by detecting the antigen‐antibody complexes using enhanced chemiluminescence reagent. We stained the membrane with 0.02% methylene blue to affirm that the same amount of DNA was spotted.

### 2.7 µCT analysis

µCT analysis was performed using high‐resolution µCT as previously described.^33^ The scanner was set as 153 μA, 65 kV, with 15 µm per pixel. The parameters of distal femoral metaphyseal trabecular bone were analyzed using software as previously reported.^33^ Trabecular bone volume per tissue volume (Tb.BV/TV), trabecular number (Tb.N), trabecular separation (Tb.Sp), and trabecular thickness (Tb.Th) were evaluated.

### 2.8 Immunofluorescence

Femurs were fixed in 4% paraformaldehyde (PFA) overnight at 4°C, decalcified with 0.5 mol/L EDTA (pH 7.45) at 4°C for 48 h, and embedded in paraffin. 4‐μm‐thick bone slides were blocked in 5% BSA for 60 min at 25°C after antigen retrieval and then stained with primary antibodies to mouse Nestin (Millipore, MAB353) and Alkbh1 (Abcam, ab128895) at 4°C overnight. The next day, the bone sections were stained using the fluorescence‐conjugated secondary antibodies at 25°C for 60 min and the nucleuses were stained using DAPI.

Cultured BMSCs were fixed in 4% PFA for 15 min, blocked in 5% BSA for 60 min at 25°C, and incubated with primary antibodies to mouse Nestin (Millipore, MAB353) and Alkbh1 (Abcam, ab128895) at 4°C overnight. After that, the cells were stained using the fluorescence‐conjugated secondary antibodies at 25°C for 60 min and the nucleuses were stained using DAPI.

### 2.9 Histochemistry and immunohistochemistry

Histochemistry and immunohistochemistry staining was conducted as previously described.^34^, ^35^, ^36^ Femurs were fixed with 4% PFA at 4°C for 24 h, decalcified in 10% EDTA (pH 7.4) at 4°C for 3 weeks, and embedded in paraffin. 4‐μm‐thick bone sections were processed for staining. We performed HE (hematoxylin‐eosin) staining using a standard protocol. For immunohistochemistry staining, 4‐μm‐thick bone sections were blocked with 5% BSA for 60 min at 25°C after antigen retrieval and stained using primary antibody to mouse osteocalcin (Takara M173) at 4°C overnight. After that, the immunoactivity was detected using an HRP‐streptavidin detection system (Dako). The nucleuses were counterstained with hematoxylin.

### 2.10 Calcein double‐labeling assay

Calcein double‐labeling assay was conducted as previously described.^37^, ^38^ Briefly, mice were treated with calcein (25 mg/kg; Sigma‐Aldrich) by intraperitoneal injection at 8 days and 2 days before euthanasia. The bones were fixed with 70% ethanol. Undecalcified bone sections were made to evaluate the trabecular bone formation. Four random fields of each distal femur were used to quantify the trabecular bone apposition rate (MAR).

### 2.11 Chromatin immunoprecipitation (ChIP) assays

For ChIP sequencing, mouse primary hepatocytes were isolated as previously described^39^ and transfected with mouse Alkbh1‐3xFlag‐plasmid (OBiO Technology Corporation). We performed ChIP assay with SimpleChip Kit (Cell Signaling Technology; 9003) following the manufacturer's protocols as previously described.^34^ Briefly, mouse primary hepatocytes transfected with Alkbh1‐3xFlag‐plasmid were cross‐linked (1% formaldehyde, 10 min) and the nuclei were isolated, lysed, and sheared to 100‐ to 500‐bp fragments by sonication. Flag antibody (Cell Signaling Technology, 4202) was applied to immunoprecipitate the relevant protein‐DNA complex. After washing, the immunoprecipitated complex was eluted and de‐crosslinked. ChIP DNA was extracted, purified, and then used to prepare sequencing libraries using the VAHTS Universal DNA Library Prep Kit for Illumina V3 (Catalog NO. ND607, Vazyme) and sequenced on Novaseq 6000 sequencer (Illumina).

ChIP‐qPCR assay was performed to validate the binding of Alkbh1 on optn promoter in BMSCs and detect the changes of N6‐mA levels on optn promoter after Alkbh1 knockout. The procedures were similar to what has been described in ChIP‐seq assay. The chromatin isolated from Alkbh1‐3xFlag‐plasmid transfected BMSCs, Alkbh1 knockout BMSCs, and control BMSCs were processed as described above and immunoprecipitated by Flag antibody (Cell Signaling Technology, 4202), N6‐mA antibody (Synaptic Systems, 202‐003), or IgG antibody (2729; included in SimpleChip Kit), respectively. The ChIP DNA was purified and quantified using qRT‐PCR with primers to detect optn promoter (Table S1).

### 2.12 Statistical analyses

All data are expressed as mean ± SEM. We used two‐tailed Student's t test to compare two groups. One‐way ANOVA was employed for the comparison of multiple groups. Differences were considered to be significant when *p* < 0.05. Sample sizes were chosen on the basis of the previous experience. No animals were excluded from the study. All the samples were randomly assigned, and no blinding was used.

### 2.13 Study approval

All animal studies were reviewed and approved by the Animal Care and Use Committees of the Department of Laboratory Animals of Central South University.

## 3 RESULTS

### 3.1 Alkbh1 expression in BMSCs declines during aging

**FIGURE 1:**
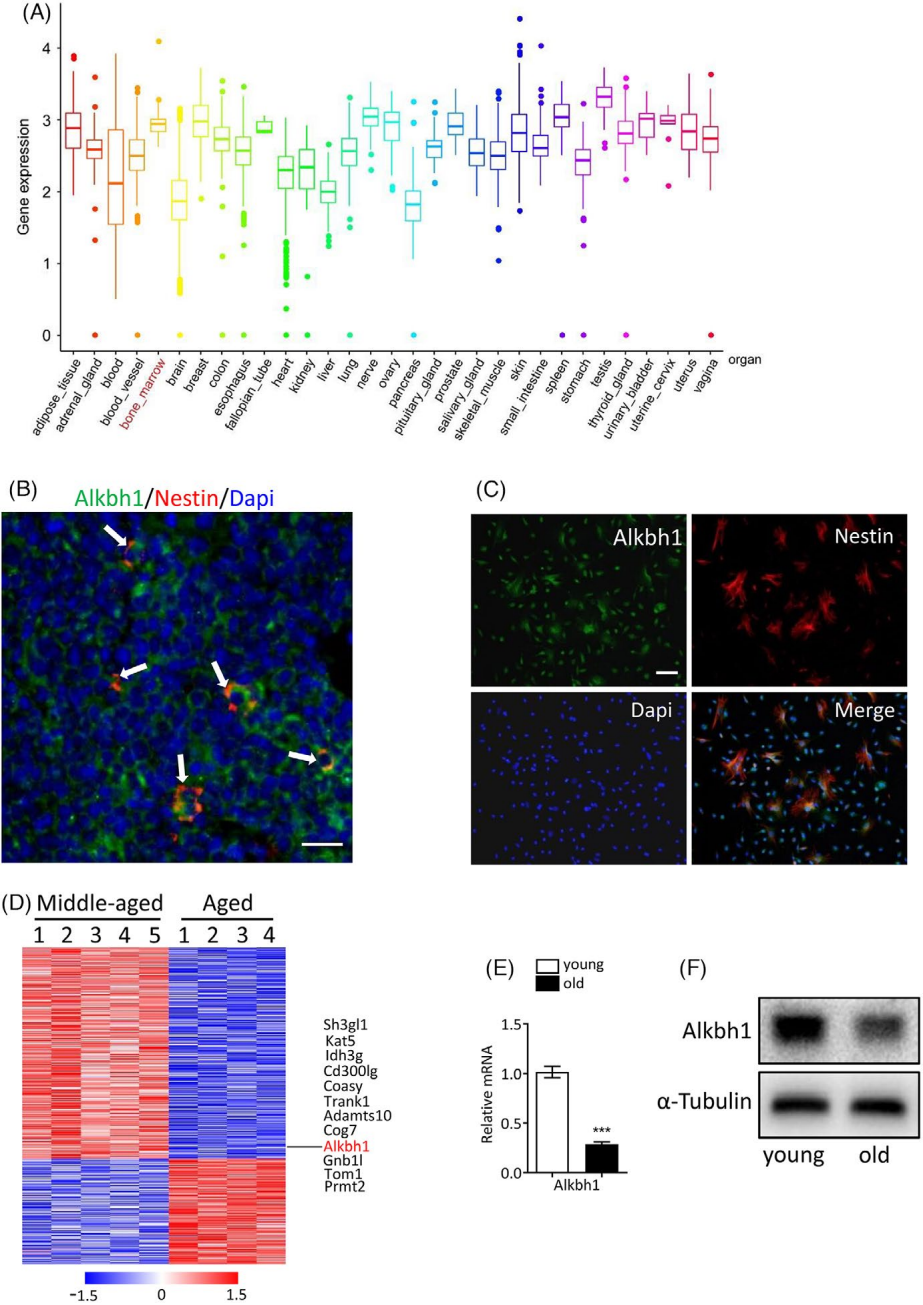
Alkbh1 expression in BMSCs declines during aging. (A) Alkbh1 expression pattern in 31 human normal tissues from GTEx. (B) Immunofluorescence reveals colocalization of Aklbh1 (green) and nestin (red) in bone marrow of 3‐month‐old mice. Scale bar: 50 μm. (C) Immunofluorescence shows overlapping of Aklbh1 (green) and nestin (red) in cultured bone marrow cells. Scale bar: 100 μm. (D) Heatmap of top 500 DEGs in microarray data (GSE35955). (E and F) qRT‐PCR (E) and western blot (F) analysis of Alkbh1 expression in BMSCs from young (3‐month‐old) and old (18‐month‐old) mice (*n* = 3). Data are presented as mean ± SEM. ***p < 0.001, Student's *t* test

Firstly, we analyzed the Alkbh1 expression pattern in human normal tissues from GTEx. Among the 31 tissues, Alkbh1 is relatively highly expressed in the bone marrow (Figure 1A). Previous studies demonstrated that nestin‐positive cells in bone marrow represent a subpopulation of BMSCs which form haematopoietic stem cell (HSC) niche.^40^, ^41^, ^42^ Our immunofluorescent staining results showed colocalization of Aklbh1 and nestin in a portion of bone marrow cells of 3‐month‐old mice in vivo (Figure 1B). We further validated the colocalization of Aklbh1 and nestin in a subgroup of bone marrow cells cultured in vitro (Figure 1C). By analyzing the microarray data (GSE35955) published by Benisch et al.,^43^ we found that Aklbh1 expression was decreased in BMSCs from aged people compared with that from middle‐aged controls (Figure 1D). In addition, we found that the expression of Aklbh1 reduced significantly in BMSCs from old mice (18‐month‐old) in comparison with that from young mice (3‐month‐old) as examined by qRT‐PCR analysis and western blot assay (Figure 1E,F).

### 3.2 Deletion of Alkbh1 in BMSCs leads to impaired osteogenic and enhanced adipogenic differentiation

**FIGURE 2:**
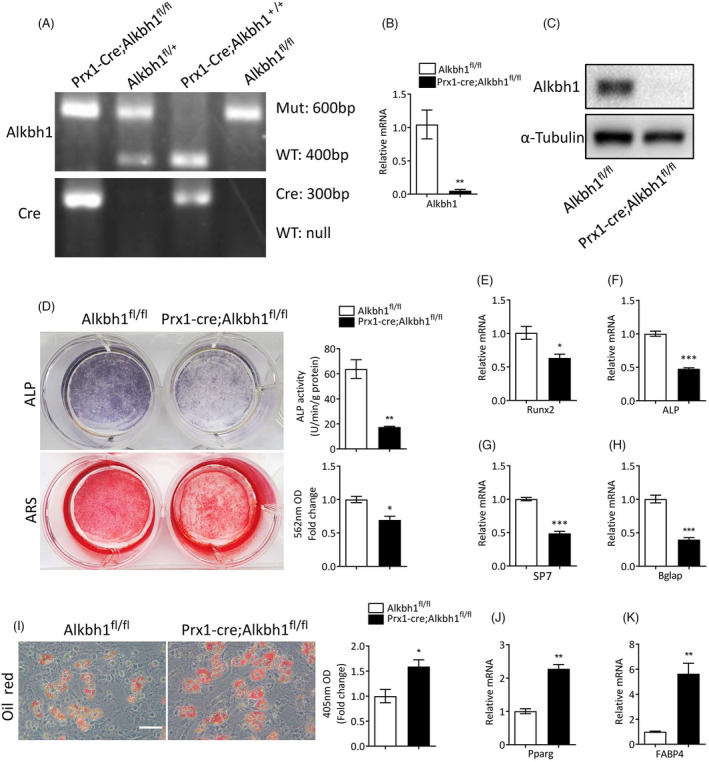
Deletion of Alkbh1 in BMSCs results in impaired osteogenic and enhanced adipogenic differentiation. (A) Representative images of PCR genotyping of transgenic mice. (B and C) qRT‐PCR (B) and western blot (C) analysis of Alkbh1 expression in BMSCs from Prx1‐Cre; Alkbh1^fl/fl^ and Alkbh1^fl/fl^ mice (*n* = 3). (D) Representative images and quantification of ARS and ALP staining of BMSCs from Prx1‐Cre; Alkbh1^fl/fl^ and Alkbh1^fl/fl^ mice under osteogenic differentiation (*n* = 3). (E–H) qRT‐PCR analysis reveals decreased transcription of Runx2, ALP, SP7, and Bglap in BMSCs from Prx1‐Cre; Alkbh1^fl/fl^ mice under osteogenic differentiation (*n* = 3). (I) Representative images and quantification of oil red O staining of BMSCs from Prx1‐Cre; Alkbh1^fl/fl^ and Alkbh1^fl/fl^ mice under adipogenic differentiation (*n* = 3). Scale bar: 50 μm. (J and K) qRT‐PCR analysis reveals increased transcription of Pparg and Fabp4 in BMSCs from Prx1‐Cre; Alkbh1^fl/fl^ mice under adipogenic differentiation (*n* = 3). Data are presented as mean ± SEM. *p < 0.05; **p < 0.01; ***p < 0.001, Student's *t* test

In order to study the role of Alkbh1 in BMSCs lineage commitment, we crossed Prx1‐Cre mice with Alkbh1 flox (Alkbh1^fl/+^) mice to generate BMSC‐specific Alkbh1 knockout mice (Prx1‐Cre; Alkbh1^fl/fl^) and the littermate controls (Alkbh1^fl/fl^) (Figure 2A). Knockout efficiency of Alkbh1 in BMSCs was confirmed by qRT‐PCR and western blot analysis (Figure 2B,C). BMSCs isolated from Prx1‐Cre; Alkbh1^fl/fl^ exhibited less ALP activity and calcium mineralization under osteogenic induction (Figure 2D). Consistently, deletion of Alkbh1 in BMSCs significantly reduced the mRNA level of osteogenic markers, including Runx2, ALP, SP7 (osterix), and Bglap (2E–H). On the contrary, knockout of Alkbh1 in BMSCs significantly promoted adipogenic differentiation as examined by oil red O staining (Figure 2I). In addition, depletion of Aklbh1 in BMSCs increased the expression of adipogenic makers, including Pparg and FABP4 as evidenced by qRT‐PCR (Figure 2J,K).

### 3.3 Conditional knockout of Alkbh1 in BMSCs results in reduced bone mass and increased marrow adiposity

**FIGURE 3:**
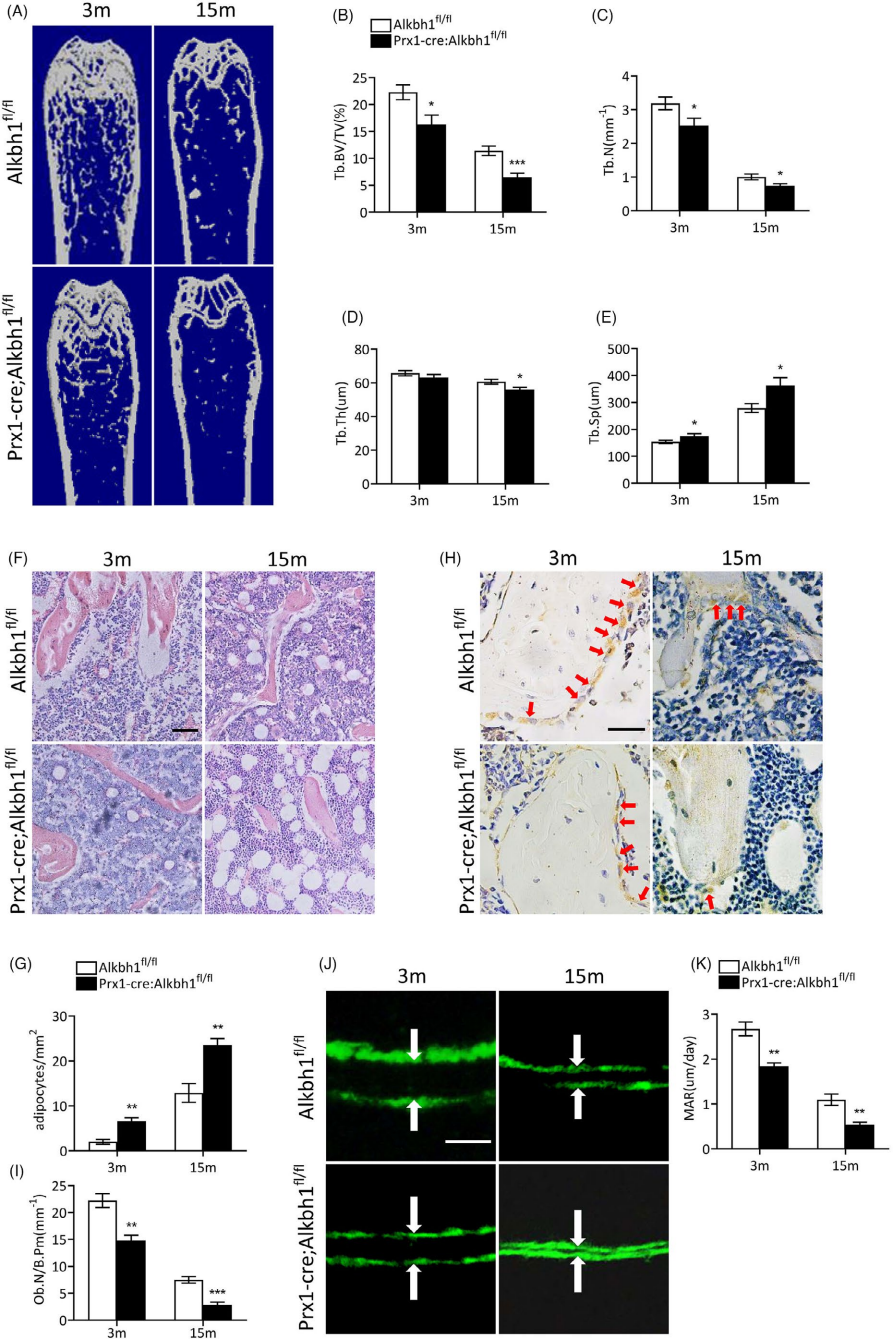
Knockout of Alkbh1 in BMSCs results in reduced bone mass and increased marrow adiposity. (A–E) Representative μCT images and quantitative analysis of femurs from 3‐month‐old and 15‐month‐old male mice (*n* = 6–8). (F and G) Representative images of HE staining (F) and quantification (G) of the number of adipocytes in femoral (*n* = 5). Scale bar: 100 μm. (H and I) Representative images of immunohistochemical staining (H) and quantitative analysis (I) of osteocalcin‐positive cells in femora (*n* = 5). Scale bar: 50 μm. (J and K) Representative images of calcein double labeling of trabecular bone (J) and quantitative analysis of MAR (K) (*n* = 5). Scale bar: 20 μm. Data are presented as mean ± SEM. *p < 0.05; **p < 0.01; ***p < 0.001, Student's *t* test

We next examined whether Aklhb1 plays an important part in osteoporosis by analyzing bone phenotypes of femurs from Prx1‐Cre; Alkbh1^fl/fl^ mice and Alkbh1^fl/fl^ littermate controls. Micro‐CT analysis of the distal femurs showed that Tb.BV/TV and Tb.N were significantly decreased in 3‐month‐old and 15‐month‐old Prx1‐Cre; Alkbh1^fl/fl^ mice in comparison with their Alkbh1^fl/fl^ littermates (Figure 3A–C). While 3‐month‐old Prx1‐Cre; Alkbh1^fl/fl^ mice and Alkbh1^fl/fl^ mice had comparable Tb.Th, 15‐month‐old Prx1‐Cre; Alkbh1^fl/fl^ mice had significantly reduced Tb.Th compared with Alkbh1^fl/fl^ littermates (Figure 3D). On the contrary, Tb.Sp increased in 3‐month‐old and 15‐month‐old Prx1‐Cre; Alkbh1^fl/fl^ mice compared with Alkbh1^fl/fl^ littermates (Figure 3E). Histomorphometric analysis revealed that 3‐month‐old and 15‐month‐old Prx1‐Cre; Alkbh1^fl/fl^ mice had significantly higher number of adipocytes and reduced number of osteoblasts than Alkbh1^fl/fl^ littermates (Figure 3F–I). Moreover, 3‐month‐old and 15‐month‐old Prx1‐Cre; Alkbh1^fl/fl^ mice had lower mineral apposition rate (MAR) compared with their Alkbh1^fl/fl^ littermates (Figure 3J,K). Together, these results indicated that BMSC‐specific Alkbh1 knockout mice exhibited reduced bone mass and increased marrow fat, suggesting aberrant fate decisions of BMSCs.

### 3.4 Loss of Alkbh1 inhibits the transcription of optn

**FIGURE 4:**
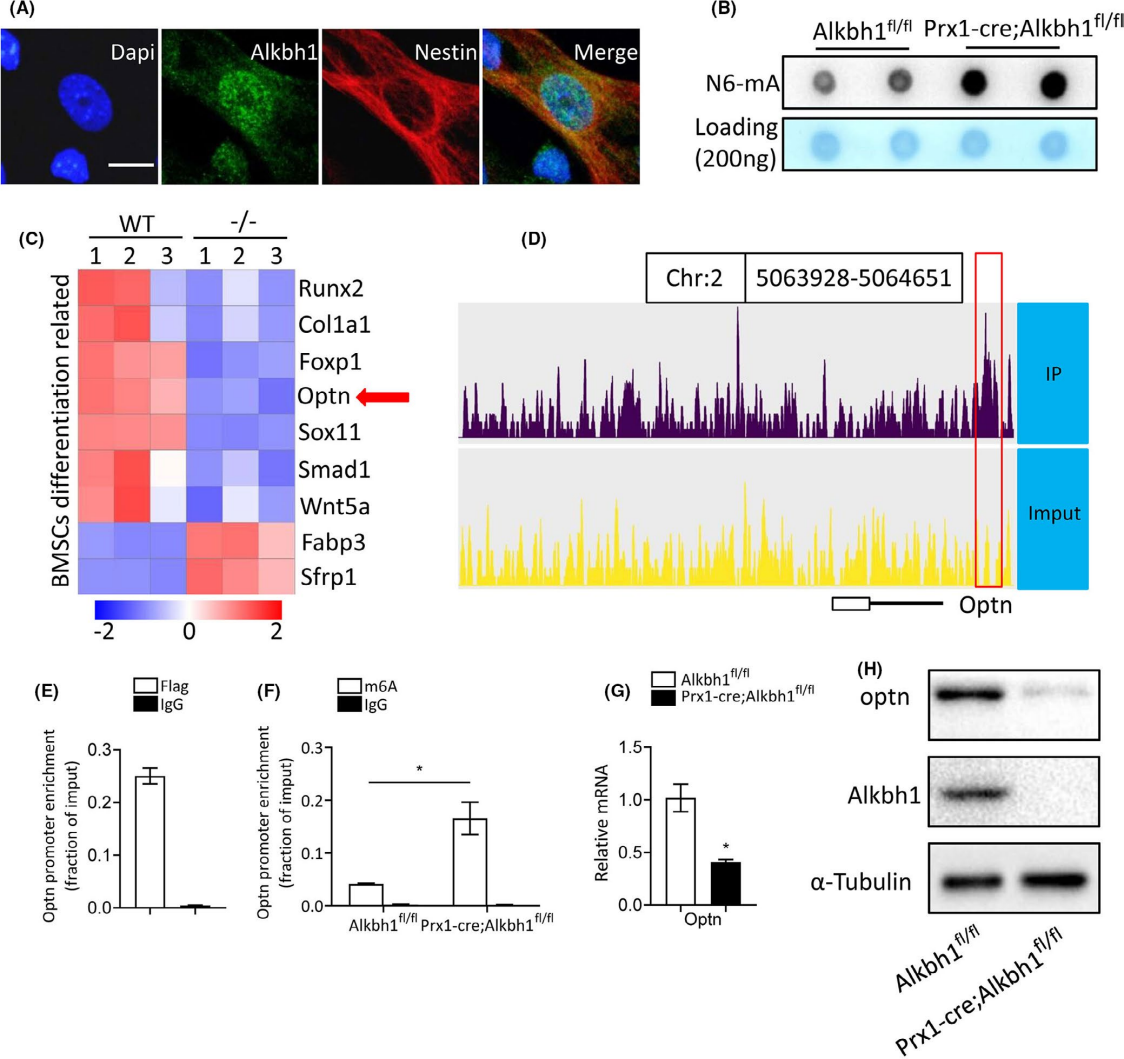
Loss of Alkbh1 inhibits the transcription of optineurin. (A) Immunofluorescence reveals that Alkbh1 protein predominantly localizes in the nucleus of mouse BMSCs. DAPI is blue, Aklbh1 is green, and nestin is red. Scale bar: 25 μm. (B) Dot blot analysis shows increased genomic DNA 6mA levels in BMSCs from Prx1‐Cre; Alkbh1^fl/fl^ mice. (C) Heatmap of selected genes associated with BMSCs differentiation (GSE30561). (D) ChIP‐seq profile shows Alkbh1‐Flag ChIP‐seq enrichments at the optn promoter region. (E) ChIP‐qPCR for Flag. Alkbh1‐Flag binds to optn promoter region (*n* = 3). (F) ChIP‐qPCR for N6‐mA. Knockout of Alkbh1 increases N6‐mA enrichment on optn promoter region (*n* = 3). (G and H) qRT‐PCR (G) and western blot (H) analysis of optn expression (*n* = 3). Data are presented as mean ± SEM. *p < 0.05, Student's *t* test

The cellular location of Alkbh1 has been a controversial issue. Ougland et al. found that human Alkbh1 is predominantly located in the nucleus of human ESCs,^44^ while Muller and his colleagues reported that human Alkbh1 is primarily in the mitochondria.^45^ Our immunofluorescence staining results revealed that mouse Alkbh1 is mainly in the nucleus of mouse BMSCs, which is consistent with its function as a DNA N6‐mA demethylase (Figure 4A). To further verify that Alkbh1 is a DNA N6‐mA demethylase, DNA dot blot assay was performed using N6‐mA antibody in Alkbh1 knockout and control BMSCs. The result showed that the levels of N6‐mA in genomic DNA of Alkbh1 knockout BMSCs increased significantly compared with that of the control BMSCs (Figure 4B), which is consistent with the previous studies.^24^, ^27^ By analyzing the microarray data (GSE30561) published by Ougland et al.,^44^ we found that optn was significantly reduced in the mouse ESCs lacking the Alkbh1 (Figure 4C). Optn has been reported to regulate BMSCs fate decisions and loss of optn leaded to accelerated bone loss and marrow fat accumulation.^46^ Moreover, our Chip sequencing result showed that Alkbh1‐Flag could bind to the promoter region of optn (Figure 4D). We also verified the binding of Alkbh1‐Flag to the promoter region of optn in BMSCs using Chip‐qPCR (Figure 4E). Furthermore, knockout of Alkbh1 increased N6‐mA saturation on the promoter region of optn as examined by Chip‐qPCR (Figure 4F). As expected, the mRNA and protein level of optn was reduced in Alkbh1 knockout BMSCs compared with the control BMSCs (Figure 4G,H). Together, these findings indicated that Alkbh1 might regulate BMSCs lineage allocation by mediating DNA N6‐mA modification on the promoter region of optn and affecting the transcription of optn.

### 3.5 Overexpression of optn partially rescues the abnormal lineage allocation of Alkbh1 knockout BMSCs

**FIGURE 5:**
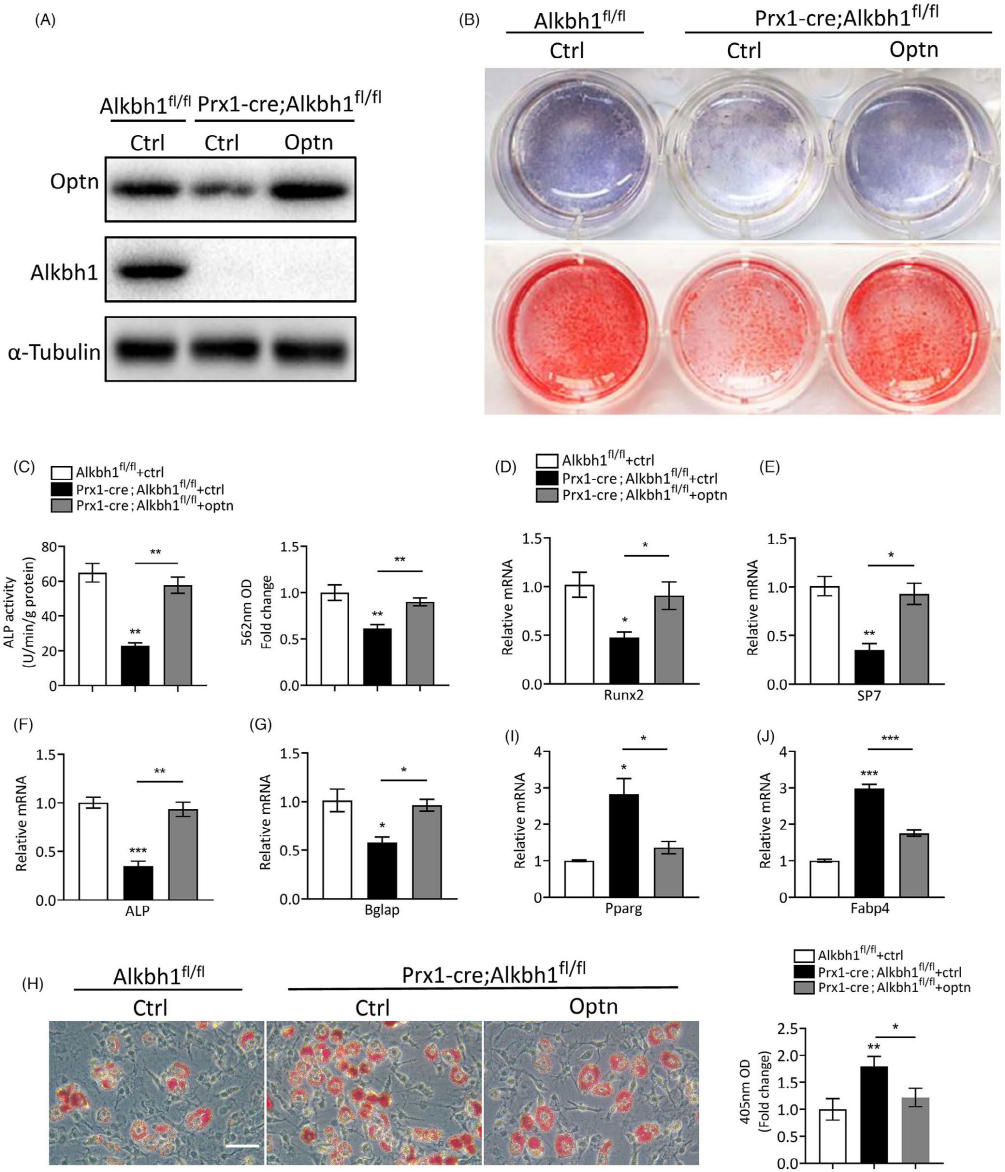
Overexpression of optn rescues the abnormal lineage allocation of Alkbh1 knockout BMSCs. (A) Western blot analysis of optn and Alkbh1. (B and C) Representative images (B) and quantification (C) of ARS and ALP staining (*n* = 3). (D–G) qRT‐PCR analysis of the transcription of Runx2, ALP, SP7, and Bglap in BMSCs under osteogenic differentiation (*n* = 3). (H) Representative images and quantification of oil red O staining of BMSCs under adipogenic differentiation (*n* = 3). Scale bar: 50 μm. (I and J) qRT‐PCR analysis the transcription of Pparg and Fabp4 in BMSCs under adipogenic differentiation (*n* = 3). Data are presented as mean ± SEM. *p < 0.05; **p < 0.01; ***p < 0.001, one‐way ANOVA

To further testify whether reduced optn expression mediated the aberrant lineage allocation of Alkbh1 knockout BMSCs, we transfected optn plasmid or the empty vector into Alkbh1 knockout BMSCs. The protein level of optn was successfully restored in Alkbh1 knockout BMSCs by transfection of optn plasmid as examined by western blot (Figure 5A). Overexpression of optn increased the ALP activity and calcium mineralization under osteogenic induction (Figure 5B,C). In addition, the mRNA levels of osteogenic markers of Alkbh1 knockout BMSC were also upregulated after optn transfection (Figure 5D–G). Moreover, optn overexpression attenuated the increased adipogenic differentiation of Alkbh1 knockout BMSC as examined by oil red O staining (Figure 5H). Consistently, qRT‐PCR analysis showed reduced expression of adipogenic markers of Alkbh1 knockout BMSC after optn transfection (Figure 5I,J).

### 3.6 Restoring Alkbh1 expression in BMSCs attenuates bone loss and marrow fat accumulation in aged mice

**FIGURE 6:**
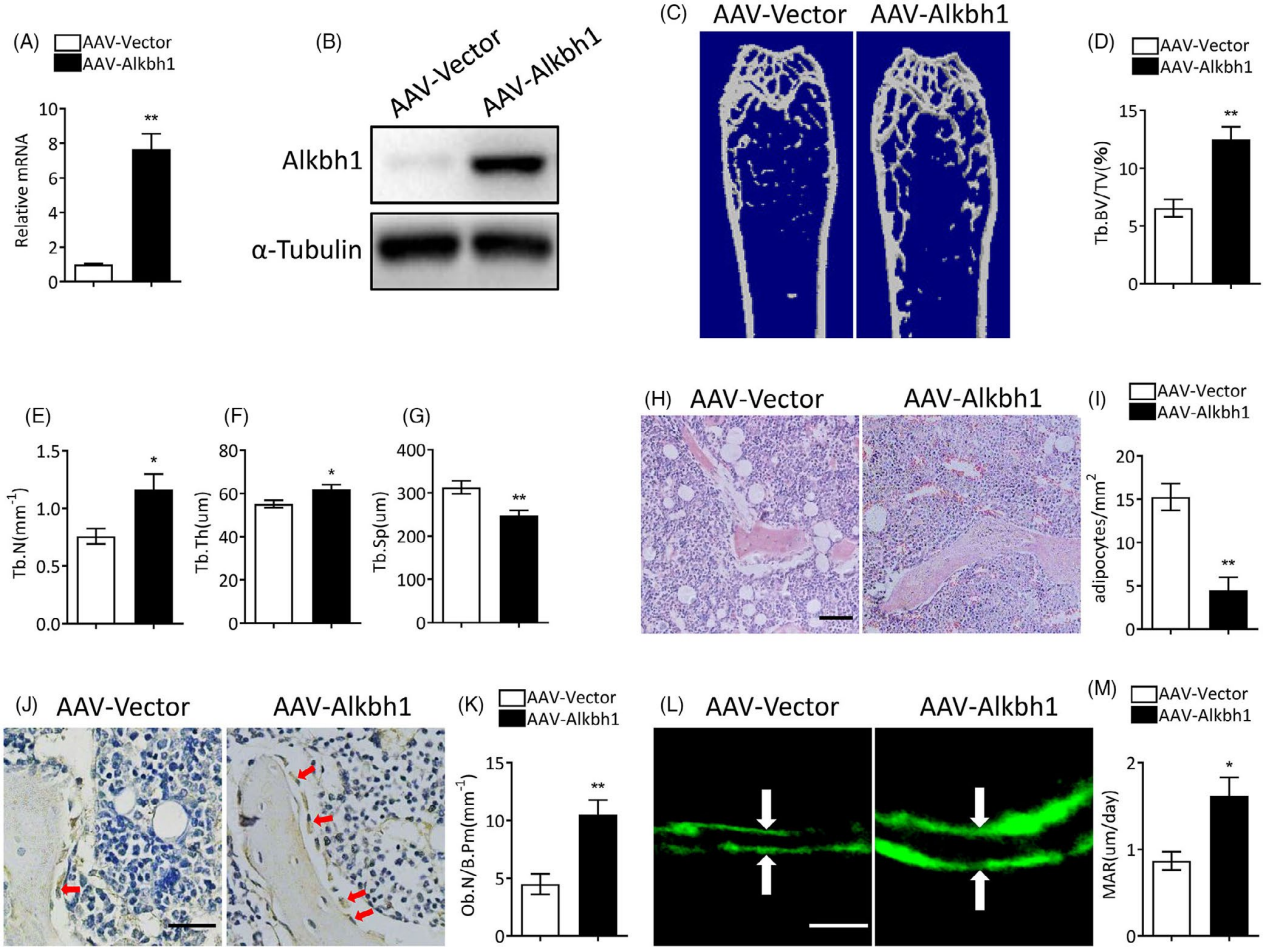
Overexpression of Alkbh1 attenuates bone loss and marrow fat accumulation in aged mice. (A and B) qRT‐PCR (A) and western blot (B) analysis of Alkbh1 expression (*n* = 3). (C–G) Representative μCT images and quantitative analysis of distal femurs (*n* = 5). (H and I) Representative images of HE staining (H) and quantification (I) of the number of adipocytes in femoral (*n* = 5). Scale bar: 100 μm. (J and K) Representative images of immunohistochemical staining (J) and quantitative analysis (K) of osteocalcin‐positive cells in femora (*n* = 5). Scale bar: 50 μm. (L and M) Representative images of calcein double labeling of trabecular bone (L) and quantitative analysis of MAR (M) (*n* = 5). Scale bar: 20 μm. Data are presented as mean ± SEM. *p < 0.05; **p < 0.01, Student's *t* test

In consideration of the pathologic role of Alkbh1 deficiency in triggering osteoporosis, we next investigated whether Alkbh1 overexpression could alleviate age‐related osteoporosis. We injected AAV‐Alkbh1 and control AAV into the bone marrow of 15‐month‐old mice via intra‐bone marrow injection. Alkbh1 expression in BMSCs of the mice infected with AAV‐Alkbh1 was much higher than that in the control group as examined by RT‐qPCR and western blot (Figure 6A,B). Micro‐CT analysis revealed that Tb.BV/TV, Tb.N, and Tb.Th were significantly increased, while Tb.SP was markedly decreased in mice infected with AAV‐Alkbh1 in comparison with that in the control mice (Figure 6C–G). Histomorphology analysis showed increased number of osteoblasts and reduced marrow fat in mice infected with AAV‐Alkbh1 compared with the control group (Figure 6H–K). Additionally, mice infected with AAV‐Alkbh1 had higher MAR compared with the control mice (Figure 6L,M). Together, these findings suggested that restoring Alkbh1 in aged mice could alleviate age‐related bone loss.

## 4 DISCUSSION

Epigenetic regulation, such as DNA methylation, was reported to play an important part in the differentiation of stem cells.^47^ Although DNA 5mC is a widely recognized DNA methylation, DNA m6A modification has been found to be a novel type of DNA methylation and Alkbh1 is identified as demethylase for DNA m6A.^20^, ^23^, ^24^ Alkbh1‐mediated DNA N6‐mA modification has extensive effects on homeostasis, and any disturbance of DNA m6A levels may lead to dysfunction or disease. Dysregulation of DNA m6A promoted tumorigenesis,^23^ enhanced the progression of atherosclerotic plaques,^48^ disrupted the ESC differentiation,^20^ inhibited skeletal muscle differentiation,^49^ as well as affected MSC osteogenic differentiation and vascular calcification.^27^, ^28^ In the present study, by analyzing the expression profile of Alkbh1 in GTEx, we found that Alkbh1 is relatively highly expressed in bone marrow. In addition, we showed decreased Alkbh1 expression in BMSCs from aged people and old mice. Our in vivo and in vitro studies showed that conditional deletion of Alkbh1 in BMSCs impaired cell lineage specification and leaded to accelerated bone aging phenotypes featured by decreased bone mass and increased marrow adiposity, indicating an effective regulation of Alkbh1‐mediated DNA m6A modification on BMSCs. Of note, overexpression of Alkbh1 attenuated bone loss and marrow fat accumulation in aged mice.

A growing number of evidences suggest that promoter‐related DNA methylation can function as a repressive mark of gene expression.^50^, ^51^ Dansranjavin and his colleagues demonstrated that increased promoter methylation results in epigenetic silence of stem cell‐related genes.^52^ In addition, Li et al. reported that Foxp1 promoter methylation increases with age, which may be the cause of reduced expression of Foxp1 in BMSC from aged mice.^41^ In our study, we found that ablation of Alkbh1 significantly increased the level of m6A in genomic DNA, which was consistent with the previous studies.^24^, ^27^ Specifically, Alkbh1 could bind to the promoter region of optn and depletion of Alkbh1 increased the m6A level on optn promoter, leading to transcriptional inhibition of optn.

Optn is identified as an autophagy receptor which plays an important role in selective autophagy. Autophagy participates in bone homeostasis, and inhibition of autophagy reduces osteogenic differentiation of BMSCs.^53^, ^54^, ^55^ Several studies demonstrated that optn mutation is closely related to Paget disease of bone (PDB), which is featured by focal increased bone turnover.^56^, ^57^, ^58^ Recently, Mizuno et al. reported that optn controls osteoblast differentiation, depletion of which impairs bone formation.^59^ Moreover, Liu et al. demonstrated that optn is critical for BMSCs fate decision and decreased optn expression during aging results in age‐related osteoporosis.^46^ However, the exact mechanism of reduced optn expression during aging is mysterious. In this study, we discovered that increased m6A level on optn promoter region due to loss of Alkbh1 contributed to reduced optn expression. In addition, we found that overexpression of optn largely reversed the trend of Alkbh1 knockout BMSCs favoring adipogenic differentiation, which further confirmed that optn as the downstream target through which Alkbh1‐mediated DNA m6A modification regulated BMSCs fate.

Previous studies reported that Alkbh1 could remove m6A from the promoter regions of bone morphogenetic protein 2 (BMP2) and activating transcription factor 4 (ATF4) to regulate osteogenic reprogramming of vascular smooth muscle cells (VSMCs) and osteogenic differentiation of human MSCs, respectively.^27^, ^28^ However, in this study, we did not find that Alkhb1 could bind to the promoter region of BMP2 and ATF4, nor did we find that the expression of BMP2 and ATF4 decreased after Alkbh1 deletion. This discrepancy may be due to cell specificity, because the cells used in those studies are human aortic smooth muscle cells and human MSCs cell lines. Meanwhile, since overexpression of optn merely achieved a partial rescue, we speculate that Alkhb1 may have other targets in BMSCs besides optn.

In summary, our study demonstrates that Alkhb1‐mediated epigenetic modification of DNA N6‐mA affects bone metabolism by regulating BMSCs fate and overexpression of Alkhb1 in aged mice can alleviate age‐related osteoporosis. Our study may provide new therapeutic strategies for the treatment of osteoporosis.

## CONFLICT OF INTEREST

The authors declare that they have no conflict of interest.

## AUTHOR CONTRIBUTIONS

Min Wang and Jian Yuan designed this study; Guang‐Ping Cai conducted most of the experiments, generated data, and wrote the manuscript; Ya‐Lin Liu, Li‐Ping Luo, and Ye Xiao interbred the mice and collected the samples; Li‐Ping Luo performed ChIP‐seq assay; Min Wang, Jian Yuan, and Tie‐Jian Jiang supervised this study, analyzed the data, and revised the manuscript.

## Supporting information

Table S1

## Data Availability

The raw data used to analyze the expression pattern and function of Alkbh1 are available in the GTEx database (https://www.gtexportal.org/) and GEO database (https://www.ncbi.nlm.nih.gov/geo/) (GSE35955 and GSE30561). Other data that support the findings of this study are available within the article or available from the authors upon request.
