# Supplementary material for: Alkbh1‐mediated DNA N6‐methyladenine modification regulates bone marrow mesenchymal stem cell fate during skeletal aging
Source: Cell Prolif. 2022 Jan 11;55(2):e13178. doi: 10.1111/cpr.13178 (PMC8828262; doi:10.1111/cpr.13178)
Supplement: Supplementary file 1 — Table S1 [file CPR-55-e13178-s001.docx]

Supplementary table 1. Primer sequence used for qRT-PCR and ChIP-qPCR.

| gene | Primer sequence (5’-3’) |
| --- | --- |
| **For qRT-PCR** |  |
| *Alkbh1*(mouse) | F: GGTGGTCAGGTTTCCTCTGAA |
|  | R: GGAGGAAGGGGTTTGGAATGAA |
| *Runx2*(mouse) | F: GAAATGCCTCCGCTGTTATG |
|  | R: AGGTGAAACTCTTGCCTCGTC |
| *ALP* (mouse) | F: CCAACTCTTTTGTGCCAGAGA |
|  | R: GGCTACATTGGTGTTGAGCTTTT |
| *Sp7*(mouse) | F: ATGGCGTCCTCTCTGCTTG |
|  | R: TGAAAGGTCAGCGTATGGCTT |
| *Bglap*(mouse) | F: AAGCAGGAGGGCAATAAGGT |
|  | R: ATGCGTTTGTAGGCGGTCTT |
| *Pparg*(mouse) | F: ATGGTTGACACAGAGATGC |
|  | R: GAATGCGAGTGGTCTTCC |
| *Fabp4*(mouse) | F: AAGGTGAAGAGCATCATAACCCT |
|  | R: TCACGCCTTTCATAACACATTCC |
| *Optn*(mouse) | F: ACAGGTGGCTACAGGTATCC |
|  | R: TGGGTGTAGGGCAGTTCTTC |
| *GAPDH* (mouse) | F: TGTGTCCGTCGTGGATCTGA |
|  | R: CCTGCTTCACCACCTTCTTGA |
| **For ChIP-qPCR** |  |
| *Optn*(mouse) | F: TCCGCGGTTCGTTAGGGATA |
|  | R: TCGGCACAGTTAAGACTGCT |

F, forward primer; R, reverse primer; qRT-PCR, quantitative real-time PCR; ChIP-qPCR, chromatin immunoprecipitation-quantitative PCR
